# Comparison of mortality according to baseline, first year, and mean albumin levels in peritoneal dialysis: a retrospective study

**DOI:** 10.1080/0886022X.2023.2176165

**Published:** 2023-02-10

**Authors:** Erdem Çankaya, Murat Altunok

**Affiliations:** Department of Nephrology, Medical Faculty, Atatürk University, Erzurum, Turkey

**Keywords:** Peritoneal dialysis, albumin level, mortality, peritonitis

## Abstract

**Background:**

The relationship between hypoalbuminemia in peritoneal dialysis (PD) and mortality, risk of peritonitis, and decreased residual renal function (RRF) is known. However, we have not encountered a comprehensive study on which of the mean albumin values, at the beginning of peritoneal dialysis, in the first year, and during the peritoneal dialysis period, provide more predictive predictions regarding mortality, peritonitis risk, and RRF reduction.

**Methods:**

A total of 407 PD patients in whom PD was initiated and followed up and PD was terminated were included in the study. Albumin levels, peritonitis, and RRF at the beginning of PD and at 3-month periods during PD were recorded.

**Results:**

In the evaluation of the patients, there was a significant relationship between mean, first-year albumin values in RRF loss (*p* = 0.001, *p* = 0.006, respectively) and peritonitis (*p* < 0.001), but no significant correlation was found with baseline albumin values (*p* = 0.213, *p* = 0.137, respectively). In the comparison of mortality ROC analysis of PD patients, a significant correlation was found with mortality at baseline, first year, and mean albumin values (*p* < 0.001). However, in the multivariate Cox regression analysis, it was determined that there was a more significant relationship between first-year albumin and mean albumin values compared to baseline albumin values (HR 0.918 [95% CI 0.302–0.528] (*p* < 0.001)), (HR 1.161 [95% CI 0.229–0.429] (*p* < 0.001)), (HR 0.081 [95% CI 0.718–1.184] (*p* = 0.525)).

**Conclusions:**

In conclusion, mean and first-year mean albumin levels provide more determinative predictions for mortality, risk of peritonitis, and maintenance of residual renal functions in peritoneal dialysis patients compared to baseline albumin.

## 1. Introduction

Peritoneal dialysis (PD) is one of the renal replacement therapies (RRTs) used for end-stage renal disease (ESRD). In different geographical regions, the rate of onset of PD in ESRD varies due to health policy differences. It has been reported that PD accounts for ∼11% of all dialysis modalities worldwide [1]. Various studies have been conducted to increase the prediction of mortality in PD and to prolong survival. Low albumin was also analyzed in studies, and it was determined that the mortality of patients with low albumin was high [2–7]. At the same time, low albumin is considered a risk factor for peritonitis in PD patients [8]. In addition, hypoalbuminemia has been associated with loss of residual renal function (RRF) [9]. There are publications reporting that increases in albumin levels during the PD process are inversely related to mortality [10–12]. Clinicians are reluctant to recommend peritoneal dialysis as an option for dialysis in patients with low serum albumin due to concerns about albumin loss with PD.

Hypoalbuminemia has been associated with increased mortality, peritonitis risk, and decreased RRF. However, there is no comprehensive study showing which of the albumin levels, i.e., at the beginning of peritoneal dialysis in the first year, mean albumin or mean albumin level in the PD process, are more determinant of this relationship. Our aim in this study was to determine which albumin level gives more decisive predictions about increased mortality, peritonitis risk, and decreased RRF.

## 2. Material and methods

### 2.1. Study design and recruitment

A total of 407 PD patients who were started and followed up by our clinic between 2005 and 2021 were examined. Patients with a history of previous renal replacement therapy (RRT), hemodialysis, or kidney transplant were excluded from the study. The mean albumin levels measured during the 3-month period before and at the beginning of PD, the albumin levels at the 3-month period during PD, and RRF were recorded. The baseline albumin level was determined by calculating the mean albumin value of the 3 months before the onset of PD. The first-year mean albumin was calculated by taking the average of the albumin value measured in 3-month periods from the beginning of PD dialysis to the end of the first year. The mean albumin level was calculated by averaging the albumin values measured at 3-month periods throughout the entire PD period. In addition, demographic and clinical characteristics of the patients, echocardiography (ECHO) reports at 6 months, the number of peritonitis, Kt/V values, and reasons for PD cessation were included in the analysis.

### 2.2. Data collection

The data of the patients were recorded from the file archive used in the follow-up and the automation system used in our hospital from the date of onset of PD. Urinary cessation was considered if the urine volume was <100 mL in 24 h. Based on 2016 ISPD guidelines, PD-related peritonitis was defined when at least two of the following are present: Abdominal pain and/or cloudy dialysis effluent, dialysis effluent white cell count >100/µL or >0.1 × 10^9^/L (after a dwell time of at least 2 h), with >50% polymorphonuclear and positive dialysis effluent culture. The peritonitis rate was calculated as the number of attacks per year recommended in ISPD 2016 [13]. Our rate of culture-negative episodes was calculated as 13% and was below the 15% stated in ISPD 2016. We validated both culture-negative and culture-positive episodes according to the diagnostic criteria and included them in the study. The overall peritonitis rate calculation included all peritonitis events, not just the first peritonitis event.

**Figure 1.**
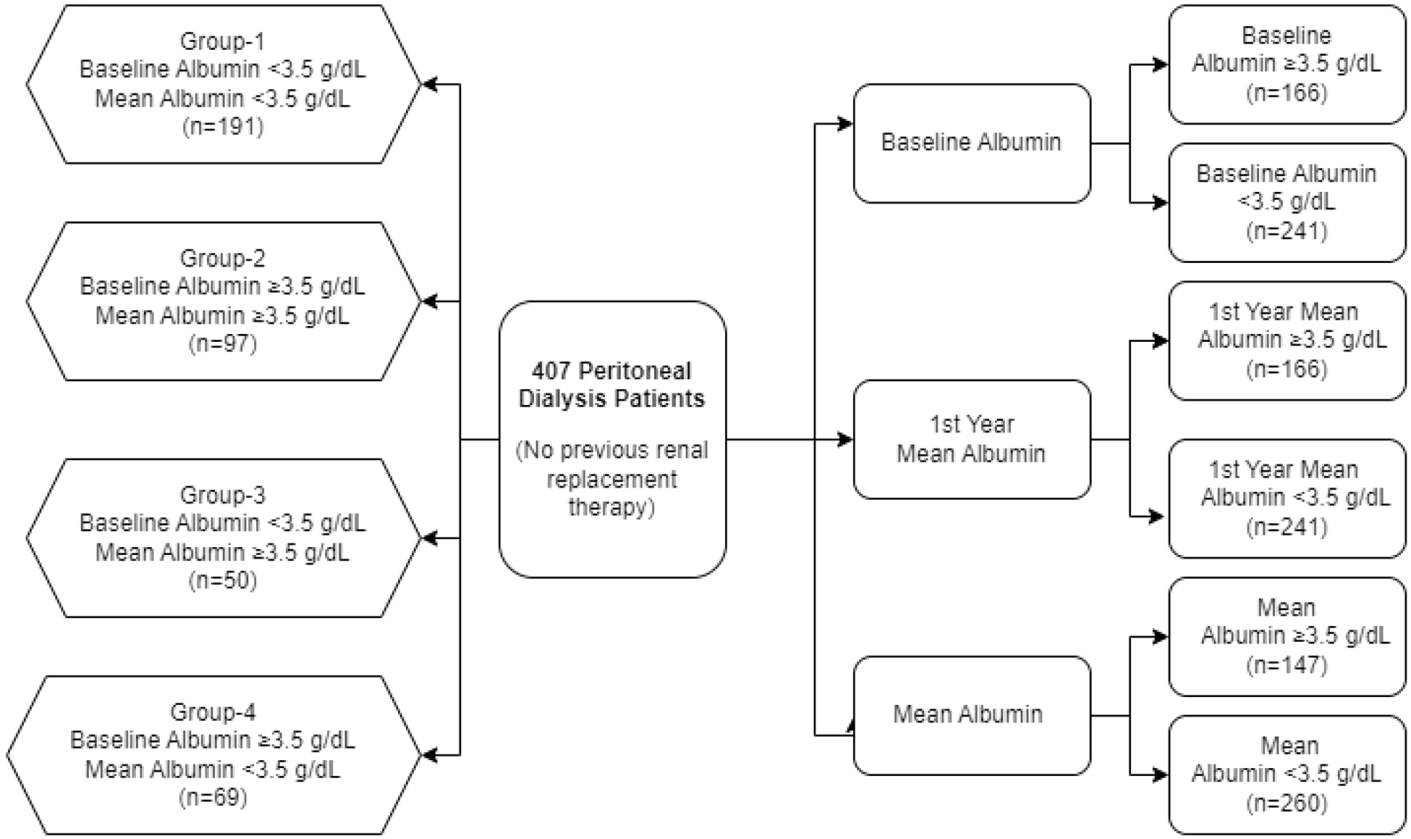
Flow chart of patients in the study group.

The patients were divided into three groups with albumin <3.5 g/dL, ≥3.5 g/dL according to the mean albumin level at baseline, first year, and during PD, and their mortality, demographic data, RRF discontinuation times, and peritonitis rates were compared. Again, baseline albumin levels and mean albumin levels of the patients were determined as baseline albumin <3.5 g/dL mean albumin <3.5 g/dL group 1, baseline albumin ≥3.5 g/dL mean albumin ≥3.5 g/dL group 2, baseline albumin <3.5 g/dL mean albumin ≥3.5 g/dL group 3, and baseline albumin ≥3.5 g/dL mean albumin <3.5 g/dL group 4, and their mortality, demographic data, RRF discontinuation times, and peritonitis rates were compared (Figure 1).

### 2.3. Pd modalities

A predialysis education program (PDEP) is a specially prepared training kit that has been used in some centers in our country. Using visual and written cards, this training kit educates CRF patients and their relatives. It has six modules, as summarized: Module 1, How do kidneys work? What is kidney failure? Which diseases cause kidney failure? Module 2, Why is diet important in kidney disease? The drugs used in kidney disease and the importance of exercise; Module 3, Introduction to the treatment of renal failure and general information about RRT; Module 4, Peritoneal dialysis; Module 5, Hemodialysis; and Module 6, Kidney transplantation.

At PD initiation, participants were assigned to one of the PD modalities, CAPD with a twin-bagged system or instrumented peritoneal dialysis (IPD). The prescription of CAPD was 4 × 1.5–2 L (body surface area, RRF determined) exchanges as long as no sign of inadequate dialysis was observed. Dialysate fluids containing (i) 1.36, 2.27, or 3.86% glucose; (ii) amino acids; or (iii) icodextrin were used according to the clinical needs of patients.

### 2.4. Statistical analysis

SPSS 20.0 (IBM SPSS Inc., Chicago, IL, USA) was used for data analysis. In the statistical analysis of the study, mean ± standard deviation for continuous variables, frequency, and percentage values for categorical variables, and interquartile range medians for skewed distributions were defined. In this study, there were no missing data except for ejection fraction, ejection fraction value, and pulmonary arterial pressure value. Pairwise deletion (available case analysis) was used to address these three parameters as missing data. General characteristics and demographic characteristics of the groups were determined by frequency (descriptive analysis: frequency analysis for a single variable) analysis. In pairwise comparisons, an independent samples *t*-test was used to compare the mean of two independent groups. One-way ANOVA (one-way analysis of variance) was used for the mean comparison of multiple independent groups. Post hoc Duncan analysis was used for multiple comparisons. The chi-square test was used to determine the relationship between categorical variables. The Pearson correlation test was used to evaluate the correlation between mean albumin and first-year mean albumin values.

The endpoint for the analysis of patient survival was death. The endpoint of the patient’s continuation of peritoneal dialysis was death, conversion to hemodialysis, and transplantation. Conversion to hemodialysis and transplantation were censored for patient survival, and mortality curves were generated using the Kaplan–Meier log-rank method. The endpoint of the patients’ RRF loss was a decrease in the amount of urine below 100 mL/day. The patient’s lack of RRF loss was censored, and graphs showing RRF loss were generated using the Kaplan–Meier log-rank method. The patients’ endpoint for the First Peritonitis Episode analysis was experiencing the first peritonitis episode. The patient’s absence of a peritonitis episode was censored, and graphs showing the peritonitis episode were generated using the Kaplan–Meier log-rank method. Receiver operating characteristic (ROC) analysis was used to determine the sensitivity and specificity of baseline albumin, first-year mean albumin, and mean albumin in predicting death.

To determine the risk factors for mortality in patients, they were grouped as older or younger than 65 years of age, having diabetes mellitus, having hypertension, having coronary artery disease, presence of urine at the end of peritoneal dialysis, decreased urine during peritoneal dialysis, experiencing peritonitis, and residual urine to be used in all models; baseline albumin and mean albumin in Model-1, baseline albumin and first-year mean albumin in Model-2, and baseline albumin and mean albumin in Model- 3 formed by Group-1, Group-2, Group-3, and Group-4. Multivariate Cox regression analysis was performed to determine risk factors for mortality in patients.

Then, to check for multicollinearity in the Cox regression models, the variance inflation factor (VIF) was calculated through the correlation (*R*) between the variables that were incorporated into the model of analysis. VIF is calculated as VIF = 1/(1 − *R*^2^). We assumed that if VIF is <2.5, the multicollinearity assumption is met. It was done by checking the correlation of the scaled Schoenfeld residual of each significant covariate with the time (PD duration), log (time), and time^2^ (Table 1) (You can reach Table 1 from the link). To assess the robustness of our results and to account for any associations we observed, we performed *E*-value analyses to assess the minimum strength of association an unmeasured confounder should have with both exposure and outcome [14]. All tests were bidirectional. Confidence intervals are given for the 5% risk of type I error.

**Table 1.** Pearson correlation for scaled schoenfeld residuals of significant covariates with time.

Covariates	Time variables		
	Time	Log (time)	Time^2^
Age Groups (<65 and ≥65) *p*-Value	0.003362 0.460	0.134995 0.397	0.000016 0.684
Presence of diabetes mellitus *p*-Value	−0.000987 0.841	−0.016938 0.920	−0.000018 0.697
Presence of hypertension *p*-Value	−0.001058 0.941	0.097024 0.580	−0.000028 0.572
Presence of coronary artery disease *p*-Value	0.003717 0.472	0.192888 0.336	0.000021 0.635
Presence of urine in peritoneal dialysis endpoint *p*-Value	−0.006728 0.153	−0.171989 0.328	−0.000055 0.181
Presence of urine reduction in the peritoneal dialysis process *p*-Value	−0.002199 0.825	−0.000217 0.999	−0.000055 0.660
Having peritonitis *p*-Value	0.011350 0.143	0.346674 0.101	0.000104 0.250
Baseline albumin *p*-Value	0.004777 0.244	0.146007 0.256	0.000037 0.391
First-year mean albumin *p*-Value	0.004919 0.322	0.079541 0.607	0.000057 0.230
Mean albumin *p*-Value	−0.000970 0.855	−0.129562 0.448	0.000012 0.779
Albumin groups *p*-Value	0.000658 0.723	0.034840 0.613	0.000007 0.637
Urine volume *p*-Value	0.000001 0.742	0.000043 0.725	0.000001 0.670

The study sample was determined to be 144 using the PASS15 program by taking *α* = 0.05 and power (1 − β) = 0.90 confidence level. We included 407 patients in the study which was more than others. *α* and (1 − *β*) values were determined by evaluating some studies carried out about PD [15,16]. A *p* ≤ 0.05 value was considered statistically significant in the entire study.

## 3. Results

**Figure 2.**
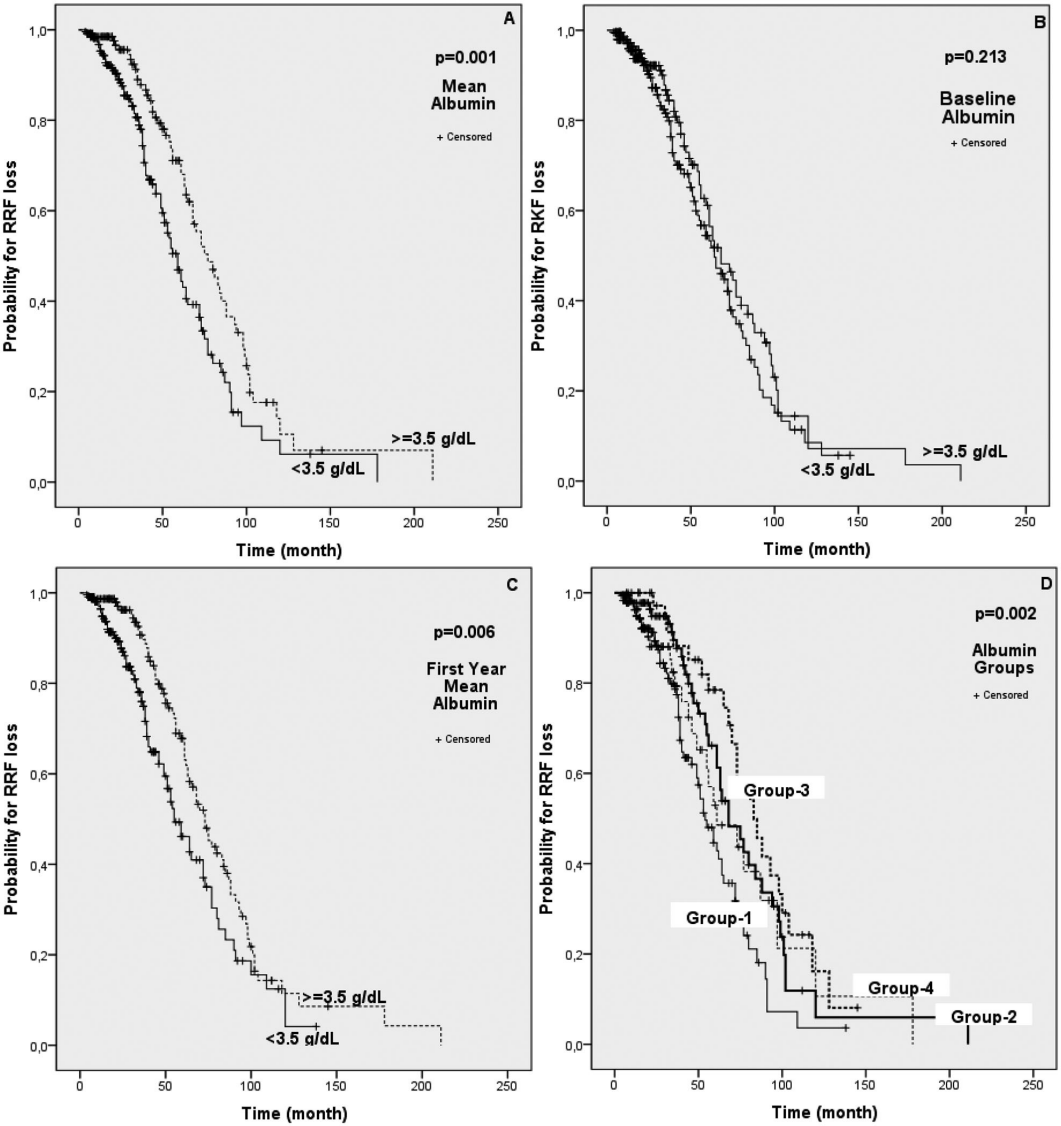
Kaplan–Meier log-rank analysis of RRF loss by albumin values and groups. (A) Analysis of RRF loss relative to the 3.5 g/dl level of mean albumin. (B) Analysis of RRF loss relative to 3.5 g/dl of baseline albumin. (C) Analysis of RRF loss relative to the 1st year mean albumin level of 3.5 g/dl. (D) Analysis of RRF loss by group-1, group-2, group-3, and group-4. *p* < 0.05 was considered statistically significant.

A total of 407 PD patients were grouped according to their mean albumin levels at baseline, first-year mean albumin levels and mean albumin values during PD (Figure 1). There was no difference between the sexes when the groups were compared. The mean age of the groups with hypoalbuminemia was higher in all groups. Diabetes mellitus, one of the primary diseases, was seen significantly more frequently in patients with hypoalbuminemia in all baseline, first-year mean, and mean albumin value groups (Table 2). Again, in all three groups, the RRF levels of those with hypoalbuminemia were significantly lower (Table 2). However, patients with hypoalbuminemia had shorter urinary withdrawal times (Table 2). In the Kaplan–Meier analysis, there was no significant difference in RRF loss between the groups with and without hypoalbuminemia according to the baseline albumin value (*p* = 0.213) (Figure 2(B)). On the other hand, it was observed that the group with hypoalbuminemia according to the mean and first-year albumin values had significant RRF loss (*p* = 0.001 and *p* = 0.006, respectively) (Figures 2(A,C)).

**Table 2.** Comparison of demographic, clinical, RRF, and peritonitis rates of peritoneal patients according to baseline, 1st-year, and mean albumin levels.

			Baseline albumin		1st year mean albumin				Mean albumin		
			≥3.5 g/dL (*n* = 166)	<3.5 g/dL (*n* = 241)	*p*	≥3.5 g/dL (*n* = 166)	<3.5 g/dL (*n* = 241)	*p*	≥3.5 g/dL (*n* = 147)	<3.5 g/dL (*n* = 260)	*p*
Gender	Female	*n* (%)	85 (51.2)	115 (47.7)	0.545	88 (53.0)	112 (46.5)	0.226	75 (51.0)	125 (48.1)	0.606
	Male	*n* (%)	81 (48.8)	126 (52.3)		78 (47.0)	129 (53.5)		72 (49.0)	135 (51.9)	
Age	≥65	*n* (%)	38 (22.9)	76 (31.5)	0.507	34 (20.5)	80 (33.2)	0.005*	26 (17.7)	88 (33.8)	0.001*
	<65	*n* (%)	128 (77.1)	165 (68.5)		132 (79.5)	161 (66.8)		121 (82.3)	172 (66.2)	
Age	Mean ± *SD*		49.11 ± 18.282	55.15 ± 16.601	0.001*	48.11 ± 17.958	55.83 ± 16.558	0.001*	46.62 ± 17.72	56.11 ± 16.507	0.001*
BMI	Mean ± *SD*		24.318 ± 5.9394	24.141 ± 5.1434	0.749	24.03 ± 5.9264	24.339 ± 5.1511	0.576	23.749 ± 5.4208	24.475 ± 5.4994	0.199
Peritoneal technique	Surgery	*n* (%)	109 (65.7)	154 (63.9)	0.752	106 (63.9)	157 (65.1)	0.833	96 (65.3)	167 (64.2)	0.914
	Percutaneous	*n* (%)	57 (34.3)	87 (36.1)		60 (36.1)	84 (34.9)		51 (34.7)	93 (35.8)	
Method of peritoneal dialysis	IPD	*n* (%)	30 (18.1)	44 (18.3)	1.000	38 (22.9)	36 (14.9)	0.050	34 (23.1)	40 (15.4)	0.061
	CAPD	*n* (%)	136 (81.9)	197 (81.7)		128 (77.1)	205 (85.1)		113 (76.9)	220 (84.6)	
PET	HP	*n* (%)	45 (27.1)	77 (32)	0.278	37 (22.3)	85 (35.3)	0.036	32 (21.8)	90 (34.6)	0.058
	HM	*n* (%)	69 (41.6)	106 (44.0)		82 (49.4)	93 (38.6)		70 (47.6)	105 (40.4)	
	LM	*n* (%)	39 (23.5)	48 (19.9)		38 (22.9)	49 (20.3)		35 (23.8)	52 (20)	
	LP	*n* (%)	13 (7.8)	10 (4.1)		9 (5.4)	14 (5.8)		10 (6.8)	13 (5)	
Kt/V	Mean ± *SD*		2.772 ± 1.2339	2.546 ± 1.1094	0.054	2.778 ± 1.2483	2.541 ± 1.0974	0.049	2.797 ± 1.3093	2.548 ± 1.0681	0.038*
Primary disease	DM	*n* (%)	30 (18.1)	83 (34.4)	0.001*	28 (16.9)	85 (35.3)	0.001*	21 (14.3)	92 (35.4)	0.001*
	HT	*n* (%)	35 (21.1)	45 (18.7)		36 (21.7)	44 (18.3)		30 (20.4)	50 (19.2)	
	CGN	*n* (%)	46 (27.7)	45 (18.7)		46 (27.7)	45 (18.7)		41 (27.9)	50 (19.2)	
	Amyloidosis	*n* (%)	8 (4.8)	22 (9.1)		10 (6)	20 (8.3)		10 (6.8)	20 (7.7)	
	KIN	*n* (%)	25 (15.1)	19 (7.9)		18 (10.8)	26 (10.8)		20 (13.6)	24 (9.2)	
	Other	*n* (%)	22 (13.3)	27 (11.2)		28 (16.9)	21 (8.7)		25 (17.0)	24 (9.2)	
Is there a DM?	Yes	*n* (%)	31 (18.7)	84 (34.9)	0.001*	29 (17.5)	86 (35.7)	0.001*	22 (15.0)	93 (35.8)	0.001*
	No	*n* (%)	135 (81.3)	157 (65.1)		137 (82.5)	155 (64.3)		125 (85.0)	167 (64.2)	
Is there HT?	Yes	*n* (%)	111 (66.9)	171 (71.0)	0.384	120 (72.3)	162 (67.2)	0.325	101 (68.7)	181 (69.6)	0.911
	No	*n* (%)	55 (33.1)	70 (29.0)		46 (27.7)	79 (32.8)		46 (31.3)	79 (30.4)	
Is there a KAH?	Yes	n (%)	30 (18.1)	40 (16.6)	0.691	32 (19.3)	38 (15.8)	0.423	26 (17.7)	44 (16.9)	0.891
	No	n (%)	136 (81.9)	201 (83.4)		134 (80.7)	203 (84.2)		121 (82.3)	216 (83.1)	
Ejection fraction	≥50	*n* (%)	74 (86.0)	91 (86.7)	1.000	84 (84.0)	81 (89.0)	0.399	74 (85.1)	91 (87.5)	0.675
	<50	*n* (%)	12 (14.0)	14 (13.3)		16 (16.0)	10 (11.0)		13 (14.9)	13 (12.5)	
EF value	Mean ± *SD*		54.97 ± 8.737	55.29 ± 7.267	0.782	54.85 ± 8.703	55.46 ± 7.046	0.596	54.9 ± 8.322	55.35 ± 7.645	0.698
PAB value	Mean ± *SD*		37.21 ± 13.3	42.44 ± 20.058	0.109	40.82 ± 15.116	39.19 ± 19.477	0.618	41.5 ± 15.496	38.76 ± 18.842	0.404
Is there urine output?	Yes	*n* (%)	153 (92.2)	220 (91.3)	0.856	153 (92.2)	220 (91.3)	0.856	134 (91.2)	239 (91.9)	0.853
	No	*n* (%)	13 (7.8)	21 (8.7)		13 (7.8)	21 (8.7)		13 (8.8)	21 (8.1)	
Urine volume (mL/day)	Mean ± *SD*		1082.83 ± 637.436	951.24 ± 600.849	0.035*	1070.18 ± 631.520	959.96 ± 606.874	**0.037**	1091.16 ± 649.38	956.15 ± 596.354	0.034*
Is there urine on the way out?	Yes	*n* (%)	99 (59.6)	152 (63.1)	0.534	88 (53.0)	163 (67.6)	0.004*	82 (55.8)	169 (65.0)	0.072
	No	*n* (%)	67 (40.4)	89 (36.9)		78 (47.0)	78 (32.4)		65 (44.2)	91 (35.0)	
Volume of urine output (mL/day)	Mean ± *SD*		400.60 ± 518.885	380.71 ± 492.899	0.696	370.10 ± 524.859	398.96 ± 488.438	0.625	408.16 ± 546.253	377.88 ± 477.770	0.560
Is there a reduction in urine volume?	Yes	*n* (%)	134 (87.6)	188 (85.5)	0.646	137 (89.5)	185 (84.1)	0.168	119 (88.8)	203 (84.9)	0.347
	No	*n* (%)	19 (12.4)	32 (14.5)		16 (10.5)	35 (15.9)		15 (11.2)	36 (15.1)	
Urine reduction volume (mL/day)	Mean ± *SD*		754.90 ± 577.106	644.09 ± 529.656	0.056	762.09 ± 562.450	639.09 ± 539.401	0.034*	762.31 ± 570.596	648.74 ± 537.500	0.056
Urine interruption time	Mean ± *SD*		39.35 ± 26.184	30.6 ± 20.21	0.041*	42.03 ± 24.129	25.95 ± 19.248	0.001*	42.48 ± 21.214	28.61 ± 23.133	0.001*
PD duration	Mean ± *SD*		43.34 ± 32.811	39.72 ± 30.326	0.252	52.89 ± 35.173	33.15 ± 25.594	0.001*	51.90 ± 35.944	35.14 ± 26.699	0.001*
Cause of PD endpoint	Exitus	*n* (%)	55 (33.1)	139 (57.7)	0.001*	52 (31.3)	142 (58.9)	0.001*	39 (26.5)	155 (59.6)	0.001*
	Peritonitis	*n* (%)	37 (22.3)	41 (17.0)		37 (22.3)	41 (17.0)		32 (21.8)	46 (17.7)	
	Transplantation	*n* (%)	38 (22.9)	21 (8.7)		39 (23.5)	20 (8.3)		39 (26.5)	20 (7.7)	
	Technical problems	*n* (%)	30 (18.1)	35 (14.5)		33 (19.9)	32 (13.3)		34 (23.1)	31 (11.9)	
	Own will	*n* (%)	6 (3.6)	5 (2.1)		5 (3.0)	6 (2.5)		3 (2.0)	8 (3.1)	
Did he/she have peritonitis?	Yes	*n* (%)	122 (73.5)	188 (78.0)	0.344	125 (75.3)	185 (76.8)	0.813	107 (72.8)	203 (78.1)	0.229
	No	*n* (%)	44 (26.5)	53 (22.0)		41 (24.7)	56 (23.2)		40 (27.2)	57 (21.9)	
Patient peritonitis rate (episode/year)			0.66	0.69		0.55	0.81		0.53	0,80	0.66
Time of first episode of peritonitis	Mean ± *SD*		23.88 ± 22.708	21.56 ± 21.135	0.811	28.59 ± 25.458	18.13 ± 17.823	0.001*	27.46 ± 24.167	19.52 ± 19.880	0.001*
Total protein	Mean ± *SD*		6.905 ± 0.6328	6.115 ± 0.836	0.001*	6.702 ± 0.7228	6.255 ± 0.8881	0.001*	6.719 ± 0.7406	6.278 ± 0.8718	0.001*
Baseline albumin	Mean ± *SD*		3.858 ± 0.3814	2.906 ± 0.4463	***0.001***	3.613 ± 0.5877	3.075 ± 0.5607	0.001*	3.639 ± 0.6035	3.099 ± 0.5567	0.001*
1st year mean albumin	Mean ± *SD*		3.603 ± 0.51454	3.0886 ± 0.55777	0.001*	3.8551 ± 0.32148	2.9149 ± 0.41039	0.001*	3.865 ± 0.34549	2.9781 ± 0.45266	0.001*
Mean albumin	Mean ± *SD*		3.534 ± 0.47042	3.0747 ± 0.52274	0.001*	3.7466 ± 0.31687	2.9283 ± 0.41047	0.001*	3.8122 ± 0.28262	2.951 ± 0.40035	0.001*
Albumin output	Mean ± *SD*		3.360 ± 0.6089	3.000 ± 0.6063	0.001*	3.5949 ± 0.5394	2.869 ± 0.5347	0.001*	3.668 ± 0.4862	2.852 ± 0.5011	0.001*
Number of albumin measurements	Mean ± *SD*					5.84 ± 0.53	5.70 ± 0.76	0.001*	18.94 ± 11.99	13.37 ± 8.90	0.001*
Triglyceride	Mean ± *SD*		185.02 ± 104.787	176.61 ± 105.788	0.429	190.95 ± 116.471	172.53 ± 96.456	0.083	190.97 ± 117.907	173.86 ± 87.207	0.116
Cholesterol	Mean ± *SD*		202.93 ± 52.629	208.55 ± 56.213	0.310	208.20 ± 51.956	204.92 ± 56.717	0.553	207.18 ± 50.827	205.74 ± 56.988	0.799
HDL	Mean ± *SD*		43.42 ± 12.289	43.48 ± 12.285	0.958	42.87 ± 11.568	43.85 ± 12.742	0.429	43.63 ± 11.094	43.35 ± 12.909	0.826
LDL	Mean ± *SD*		134.74 ± 42.082	146.74 ± 129.144	0.249	139.43 ± 40.832	143.51 ± 129.621	0.695	139.12 ± 38.945	143.39 ± 125.602	0.688
Baseline uric acid	Mean ± *SD*		6.598 ± 1.647	6.046 ± 1.4288	0.001*	6.402 ± 1.5077	6.181 ± 1.5647	0.156	6.457 ± 1.5309	6.166 ± 1.5438	0.067
Uric acid output	Mean ± *SD*		6.023 ± 1.5258	5.463 ± 1.2930	0.001*	5.824 ± 1.4395	5.600 ± 1.3984	0.117	5.823 ± 1.3203	5.617 ± 1.4674	0.159

BMI: body mass index; IPD: instrumented peritoneal dialysis; CAPD: continuous ambulatory peritoneal dialysis; PET: peritoneal equalization test; HP: high permeable; HM: high-moderate permeable; LM: low-moderate permeable; LP: low permeable; DM: diabetes mellitus; Ht: hypertension; CGN: chronic glomerulonephritis; CIN: chronic interstitial nephritis; CAD: coronary artery disease; EF: ejection fraction; PAP: pulmonary artery pressure.

*A value of *p* < 0.05 was considered statistically significant.

**Figure 3.**
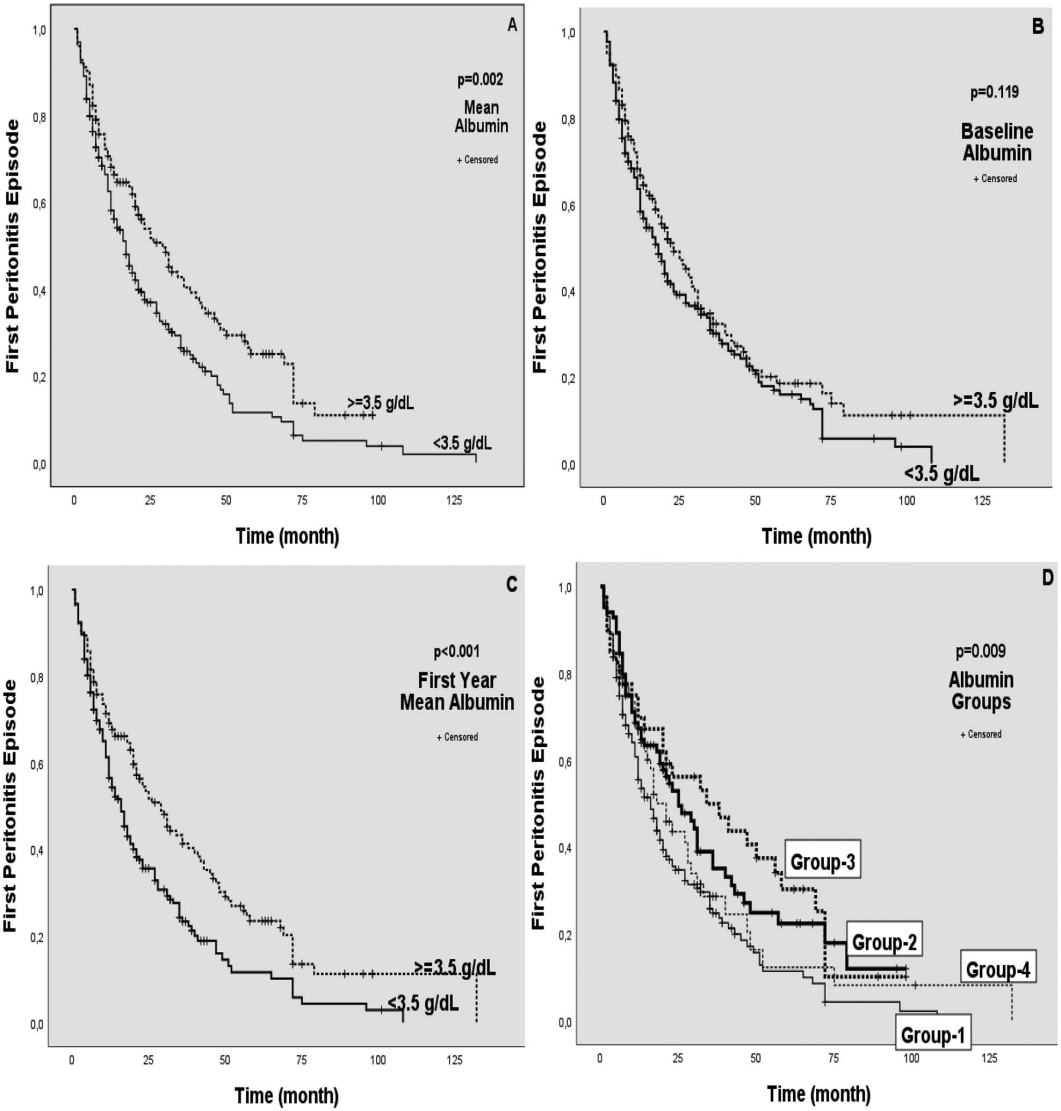
Kaplan–Meier analysis of first peritonitis episode risk by albumin values and groups. (A) Analysis of first peritonitis episode risk relative to the mean albumin level of 3.5 g/dl. (B) Analysis of first peritonitis episode risk relative to 3.5 g/dl of baseline albumin. (C) Analysis of first peritonitis episode risk based on the 1st year mean albumin level of 3.5 g/dl. (D) Analysis of first peritonitis episode risk according to group-1, group-2, group-3, and group-4. *p* < 0.05 was considered statistically significant.

When the first peritonitis rates were examined, it was determined that the group with high albumin values between the mean albumin and first-year albumin values had a lower peritonitis rate. However, this difference was not observed between the group with hypoalbuminemia at the baseline albumin level and the group with normal albumin (0.55–0.81, 0.53–0.80, 0.66–0.69) (Table 2). In the Kaplan–Meier analysis performed to evaluate the risk of peritonitis, it was determined that the group with hypoalbuminemia according to mean albumin and first-year albumin had significantly more peritonitis than the group without hypoalbuminemia (*p* = 0.002, *p* < 0.001) (Figures 3(A,C)). There was no significant difference between the group with and without hypoalbuminemia according to baseline albumin (*p* = 0.119) (Figure 3(B)).

**Figure 4.**
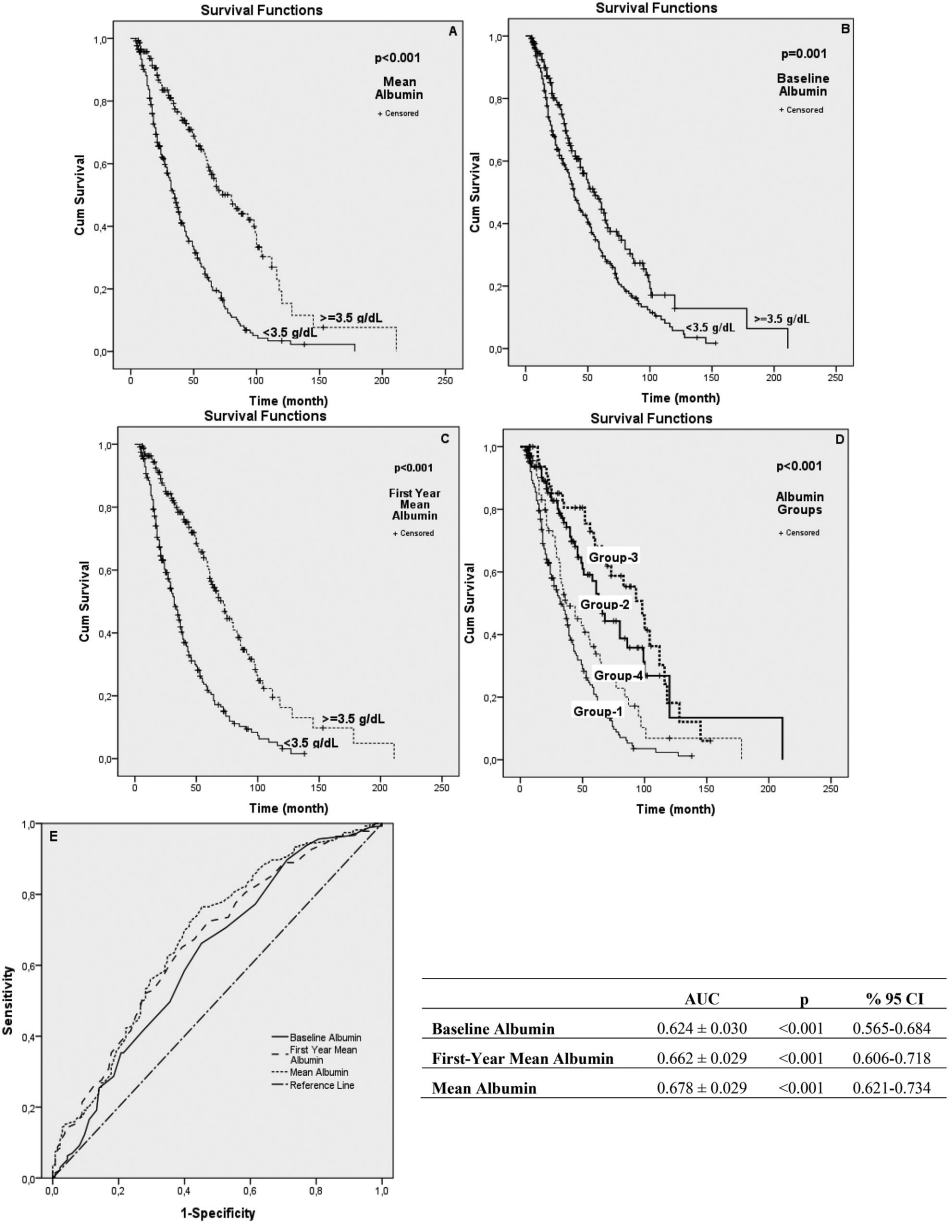
Kaplan–Meier analysis of mortality by albumin values and groups. (A) Mortality analysis based on the 3.5 g/dl level of the mean albumin. (B) Mortality analysis based on 3.5 g/dl of baseline albumin. (C) Mortality analysis based on the 1st year mean albumin level of 3.5 g/dl. (D) Mortality analysis by group-1, group-2, group-3, and group-4. (E) ROC analysis graph showing mortality estimation by baseline, mean, and 1st year mean albumin values. *p* < 0.05 was considered statistically significant.

According to the baseline albumin value, the mean duration of PD for the group with hypoalbuminemia was determined to be 30 months, and the group with normal albumin level was determined to be 35 months. The duration of PD was determined to be 25 months for the group with an albumin level <3.5 g/dL in the first year and 47.5 months for the group with an albumin level ≥3.5 g/dL. According to the mean albumin value, the duration of PD for the group with <3.5 g/dL was 27 months, and the group with albumin ≥ 3.5 g/dL was determined to be 46 months (Table 3). When the patients with hypoalbuminemia at baseline, first year, and mean albumin groups were compared, it was found that patients with hypoalbuminemia at baseline had a longer duration of peritoneal dialysis (Table 3). The total follow-up time (patient-month at risk) of patients with a baseline albumin value ≥3.5 g/dL was 56 ± 5.933 months, and it was 39 ± 2.693 months for baseline albumin levels <3.5 g/dL. According to the first-year mean albumin value, those with ≥3.5 g/dL had a mean survival time of 72 ± 5.173 months, and those with albumin levels <3.5 g/dL had a mean survival time of 32 ± 2.274 months. Average survival times were determined according to the mean albumin value of ≥3.5 g/dL to be 80 ± 9.185 months and albumin level <3.5 g/dL to be 35 ± 2.396 months (Table 3, Figure 1). In the Kaplan–Meier analysis of mortality according to albumin values and groups, it was determined that groups with hypoalbuminemia had a significant increase in mortality compared to groups without hypoalbuminemia (*p* = 0.001, *p* < 0.001, *p* < 0.001, respectively) (Figures 4(A–C)). In the ROC analysis, it was determined that baseline, first-year mean, and mean albumin values significantly affected mortality (*p* < 0.001) (Figure 4(E)).

**Table 3.** Comparison of mortality of peritoneal dialysis patients according to baseline, 1st year, and mean albumin levels.

		Median follow-up time	Case	Mean survival time	Median survival time	1. Cumulative survival rate in years	2. Cumulative survival rate in years	3. Cumulative survival rate in years	5. Cumulative survival rate in years	*p* (Log rank)
		Month (min–max)	*n*	Month ± *SD* (95% CI)	Month ± *SD* (95% CI)	% ± SE	% ± SE	% ± SE	% ± SE	
Baseline albumin	≥3.5 g/dL	35 (5–211)	92/166	70.681 ± 6.465 (58.008–83.353)	56.000 ± 5.933 (44.371–67.629)	92.5 ± 2.1	79.5 ± 3.3	64.9 ± 4.1	46.8 ± 4.6	0.001*
	<3.5 g/dL	30 (4–153)	180/241	49.296 ± 2.688 (44.027–54.565)	39.000 ± 2.693 (33.722–44.278)	88.5 ± 2.1	65.1 ± 3.2	53.7 ± 3.4	31.3 ± 3.3	
1st Year mean albumin	≥3.5 g/dL	47.5 (6–211)	89/166	79.446 ± 5.695 (68.283–90.609)	72.000 ± 5.173 (61.861–82.139)	95.0 ± 1.7	86.3 ± 2.8	77.6 ± 3.5	60.1 ± 4.4	0.001*
	<3.5 g/dL	25 (4–138)	183/241	40.936 ± 2.299 (36.430–45.442)	32.000 ± 2.274 (27.542–36.458)	87.2 ± 2.2	60.2 ± 3.3	44.1 ± 3.5	21.1 ± 3.1	
Mean albumin	≥3.5 g/dL	46 (6–211)	71/147	82.850 ± 6.591 (69.932–95.768)	80.000 ± 9.185 (61.997–98.003)	94.3 ± 1.9	85.1 ± 3.1	76.6 ± 3.8	61.1 ± 4.7	0.001*
	<3.5 g/dL	27.5 (4–178)	201/260	43.096 ± 2.432 (38.330–47.863)	35.000 ± 2.396 (30.304–39.696)	88.1 ± 2.0	62.9 ± 3.1	47.5 ± 3.3	24.2 ± 3.0	

*A value of *p* < 0.05 was considered statistically significant.

In our second evaluation, we compared Group 1, Group 2, Group 3, and Group 4 according to the baseline albumin and then the mean albumin values (Figure 1). There was no difference between the sexes of the four groups. However, group 1 was significantly older than the other groups, and the presence of the primary disease diabetes mellitus was significantly higher (Table 4). The amount of RRF was significantly higher in group 2 (*p* < 0.05) (Table 4). In addition, a significant difference was found in the loss of RRF between the groups in the Kaplan–Meier analysis (*p* = 0.002) (Figure 2(D)). RRF interruption times were significantly shorter in group 1 and group 4 with low mean albumin levels. No significant difference was found between groups 2 and 3 (Table 4). PD durations were 33.23 ± 24.484, 45.40 ± 33.623, 64.52 ± 37.254, and 40.45 ± 31,642 months, respectively (*p* < 0.001) (Table 4). The longest mean survival time was determined in group 3, and the shortest was determined in group 1 (39,033 ± 2,232 months, 82.040 ± 11.785 months, 86.230 ± 6.815 months, 54.501 ± 6.396 months, *p* < 0.001) (Table 5). Moreover, group 3 had the best mortality in the Kaplan–Meier analysis between groups 1, 2, 3, and 4, followed by groups 2, 4, and 1, and they were found to be significantly different (*p* = 0.009) (Figure 3(D)). In Kaplan–Meier analysis performed to evaluate peritonitis among groups 1, 2, 3, and 4, group 3 had the lowest peritonitis, followed by groups 2, 4, and 1 (*p* < 0.001) (Figure 4(D)).

**Table 4.** Comparison of demographic, clinical, RRF, and peritonitis rates according to baseline and mean albumin values.

			Group 1 (*n* = 191)	Group 2 (*n* = 97)	Group 3 (*n* = 50)	Group 4 (*n* = 69)	*p*
			Baseline albumin <3.5 g/dL Mean albumin <3.5 g/dL	Baseline albumin ≥3.5 g/dL Mean albumin ≥3.5 g/dL	Baseline albumin <3.5 g/dL Mean albumin ≥3.5 g/dL	Baseline albumin ≥3.5 g/dL Mean albumin <3.5 g/dL	
Gender	Female	*n* (%)	85 (44.5)	45 (46.4)	30 (60.0)	40 (58.0)	0.092
	Male	*n* (%)	106 (55.5)	52 (53.6)	20 (40.0)	29 (42.0)	
Age	≥65	*n* (%)	67 (35.1)	17 (17.5)	9 (18.0)	21 (30.4)	0.005*
	<65	*n* (%)	124 (64.9)	80 (82.5)	41 (82.0)	48 (69.6)	
Age	Mean ± *SD*		57.17 ± 16.025^b^	46.22 ± 18.323^a^	47.4 ± 16.64^a^	53.17 ± 17.56^b^	0.001*
BMI	Mean ± *SD*		24.36 ± 5.2277	23.98 ± 5.7399	23.302 ± 4.7641	24.793 ± 6.2203	0.482
Peritoneal technique	Surgery	*n* (%)	121 (63.4)	63 (64.9)	33 (66.0)	46 (66.7)	0.959
	Percutaneous	*n* (%)	70 (36.6)	34 (35.1)	17 (34.0)	23 (33.3)	
Method of peritoneal dialysis	IPD	*n* (%)	32 (16.8)	22 (22.7)	12 (24.0)	8 (11.6)	0.193
	CAPD	*n* (%)	159 (83.2)	75 (77.3)	38 (76.0)	61 (88.4)	
PET	HP	*n* (%)	68 (35.6)	23 (23.7)	9 (18.0)	22 (31.9)	0.123
	HM	*n* (%)	76 (39.8)	40 (41.2)	30 (60.0)	29 (42.0)	
	LM	*n* (%)	39 (20.4)	26 (26.8)	9 (18.0)	13 (18.8)	
	LP	*n* (%)	8 (4.2)	8 (8.2)	2 (4.0)	5 (7.2)	
Kt/V	Mean ± *SD*		2.518 ± 1.0891	2.872 ± 1.3671	2.652 ± 1.1891	2.632 ± 1.0107	0.113
Primary disease	DM	*n* (%)	76 (39.8)	14 (14.4)	7 (14.0)	16 (23.2)	0.001*
	HT	*n* (%)	35 (18.3)	20 (20.6)	10 (20.0)	15 (21.7)	
	CGN	*n* (%)	28 (14.7)	24 (24.7)	17 (34.0)	22 (31.9)	
	Amyloidosis	*n* (%)	19 (9.9)	7 (7.2)	3 (6.0)	1 (1.4)	
	KIN	*n* (%)	14 (7.3)	15 (15.5)	5 (10.0)	10 (14.5)	
	Other	*n* (%)	19 (9.9)	17 (17.5)	8 (16.0)	5 (7.2)	
Is there a DM?	Yes	*n* (%)	77 (40.3)	15 (15.5)	7 (14.0)	16 (23.2)	0.001*
	No	*n* (%)	114 (59.7)	82 (84.5)	43 (86.0)	53 (76.8)	
Is there HT?	Yes	*n* (%)	137 (71.7)	67 (69.1)	34 (68.0)	44 (63.8)	0.668
	No	*n* (%)	54 (28.3)	30 (30.9)	16 (32.0)	25 (36.2)	
Is there a KAH?	Yes	*n* (%)	35 (18.3)	21 (21.6)	5 (10.0)	9 (13.0)	0.243
	No	*n* (%)	156 (81.7)	76 (78.4)	45 (90.0)	60 (87.0)	
Ejection fraction	≥50	*n* (%)	64 (85.3)	47 (82.5)	27 (90.0)	27 (93.1)	0.519
	<50	*n* (%)	11 (14.7)	10 (17.5)	3 (10.0)	2 (6.9)	
EF value	Mean ± *SD*		54.87 ± 7.533	54.14 ± 9.078	56.33 ± 6.557	56.59 ± 7.926	0.455
Pulmonary artery pressure	<30	*n* (%)	12 (26.7)	9 (25.0)	3 (18.8)	8 (47.1)	0.268
	≥30	*n* (%)	33 (73.3)	27 (75.0)	13 (81.2)	9 (52.9)	
PAB value	Mean ± *SD*		41.27 ± 20.62	39.61 ± 13.77	45.75 ± 18.606	32.12 ± 10.937	0.137
Is there urine?	Yes	*n* (%)	177 (92.7)	91 (93.8)	43 (86.0)	62 (89.9)	0.358
	No	*n* (%)	14 (7.3)	6 (6.2)	7 (14.0)	7 (10.1)	
Urine volume (mL/day)	Mean ± *SD*		951.31 ± 592.595^a^	1163.4 ± 646.767^b^	951 ± 637.637^a^	969.57 ± 610.823^a^	0.038*
Is there urine on the way out?	Yes	*n* (%)	127 (66.5)	57 (58.8)	25 (50.0)	42 (60.9)	0.163
	No	*n* (%)	64 (33.5)	40 (41.2)	25 (50.0)	27 (39.1)	
Volume of urine output (mL/day)	Mean ± *SD*		401.57 ± 499.971	463.4 ± 579.894	301 ± 460.976	312.32 ± 406.239	0.146
Is there a reduction in urine?	Yes	*n* (%)	28 (15.8)	11 (12.1)	4 (9.3)	8 (12.9)	0.657
	No	*n* (%)	149 (84.2)	80 (87.9)	39 (90.7)	54 (57.1)	
Amount of urine reduction volume (mL/day)	Mean ± *SD*		616.95 ± 527.64	765.38 ± 591.875	755.81 ± 529.399	739.52 ± 559.137	0.115
Urine interruption time	Mean ± *SD*		25.8 ± 17.53^a^	41.66 ± 21.282^b^	43.94 ± 21.626^b^	35.65 ± 32.832^a^	0.004*
PD duration	Mean ± *SD*		33.23 ± 24.484^a^	45.40 ± 33.623^b^	64.52 ± 37.254^c^	40.45 ± 31.642^ab^	0.001*
PD outcome cause	Exitus	*n* (%)	120 (62.8)	20 (20.6)	19 (38.0)	35 (50.7)	0.001*
	Peritonitis	*n* (%)	33 (17.3)	24 (24.7)	8 (16.0)	13 (18.8)	
	Transplantation	*n* (%)	11 (5.8)	29 (29.9)	10 (20.0)	9 (13.0)	
	Technical problems	*n* (%)	23 (12.0)	22 (22.7)	12 (24.0)	8 (11.6)	
	Own will	*n* (%)	4 (2.1)	2 (2.1)	1 (2.0)	4 (5.8)	
Did he/she have peritonitis?	Yes	*n* (%)	149 (78.0)	68 (70.1)	39 (78.0)	54 (78.3)	0.461
	No	*n* (%)	42 (22.0)	29 (29.9)	11 (22.0)	15 (21.7)	
Patient peritonitis rate (year)			0.79	0.56	0.48	0.81	
Time of first episode of peritonitis	Mean ± *SD*		18.55 ± 18.464^b^	24.86 ± 22.178^ab^	32.92 ± 27.385^a^	22.44 ± 23.593^ab^	0.001*
Total protein	Mean ± *SD*		6.075 ± 0.8513^a^	6.951 ± 0.6148^b^	6.27 ± 0.763^a^	6.842 ± 0.6565^b^	0.001*
Baseline albumin	Mean ± *SD*		2.865 ± 0.4419^a^	3.936 ± 0.4442^b^	3.064 ± 0.4318^c^	3.748 ± 0.2311^d^	0.001*
1st year mean albumin	Mean ± *SD*		2.9086 ± 0.448^a^	3.9108 ± 0.32446^b^	3.776 ± 0.37036^c^	3.1703 ± 0.41064^d^	0.001*
Mean albumin	Mean ± *SD*		2.8971 ± 0.41085^a^	3.8428 ± 0.26836^c^	3.753 ± 0.30243^c^	3.1 ± 0.32887^b^	0.001*
Number of albumin measurements	Mean ± *SD*		12.74 ± 8.15^a^	16.78 ± 11.21^b^	23.12 ± 12.44^c^	15.10 ± 10.55^ab^	0.001*
Albumin output	Mean ± *SD*		2.824 ± 0.5056^a^	3.666 ± 0.4935^b^	3.672 ± 0.4768^b^	2.929 ± 0.4836^a^	0.001*
Triglyceride	Mean ± *SD*		174.61 ± 103.126	194.43 ± 119.232	184.26 ± 116.191	171.8 ± 79.18	0.421
Cholesterol	Mean ± *SD*		207.03 ± 59.023	203.47 ± 53.903	214.38 ± 43.874	202.17 ± 51.167	0.623
HDL	Mean ± *SD*		43.41 ± 12.789	43.58 ± 11.559	43.74 ± 10.244	43.19 ± 13.331	0.995
LDL	Mean ± *SD*		146.14 ± 143.92	134 ± 39.133	149.04 ± 36.967	135.78 ± 46.186	0.712
Baseline uric acid	Mean ± *SD*		6.053 ± 1.4777^a^	6.684 ± 1.6221^b^	6.018 ± 1.237^a^	6.477 ± 1.6859^ab^	0.004*
Uric acid output	Mean ± *SD*		5.486 ± 1.3574^a^	6.054 ± 1.4019^b^	5.376 ± 1.0171^a^	5.98 ± 1.6944^b^	0.001*

BMI: body mass index; IPD: instrumented peritoneal dialysis; CAPD: continuous ambulatory peritoneal dialysis; PET: peritoneal equalization test; HP: high permeable; HM: high-moderate permeable; LM: low-moderate permeable; LP: low permeable; DM: diabetes mellitus; HT: hypertension; CGN: chronic glomerulonephritis; CIN: chronic interstitial nephritis; CAD: coronary artery disease; EF: ejection fraction; PAP: pulmonary artery pressure.

a,b: means in the same column differ from each other (*p* < 0.05). Groups that do not have a common letter are different from each other.

*A value of *p* < 0.05 was considered statistically significant.

**Table 5.** Comparison of mortality according to baseline and mean serum albumin values.

		Median follow-up time	Case	Mean survival time	Median survival time	1. Cumulative survival rate in years	2. Cumulative survival rate in years	3. Cumulative survival rate in years	5. Cumulative survival rate in years	*p* (Log rank)
		Month (min–max)	*n*	Month ± *SD* (95% CI)	Month ± *SD* (95% CI)	% ± SE	% ± SE	% ± SE	% ± SE	
Overall survival		32 (4–211)	272/407	56.801 ± 2.907 (51.103–62.499)	44.000 ± 3.042 (38.038–49.962)	90.9 ± 1.4	70.8 ± 2.3	58.2 ± 2.6	37.2 ± 2.8	
Group 1	Baseline albumin <3.5 g/dL Mean albumin <3.5 g/dL	25 (4–138)	153/191	39.033 ± 2.232 (34.658–43.408)	32.000 ± 3.357 (25.420–38.580)	85.5 ± 2.6	59.3 ± 3.7	46.4 ± 3.8	20.2 ± 3.3	0.001*
Group 2	Baseline albumin ≥3.5 g/dL Mean albumin ≥3.5 g/dL	40 (6–211)	44/97	82.040 ± 11.785 (58.942–105.138)	64.000 ± 5.067 (54.068–73.932)	93.6 ± 2.5	84.0 ± 3.9	74.3 ± 4.9	53.0 ± 6.3	
Group 3	Baseline albumin <3.5 g/dL Mean albumin ≥3.5 g/dL	63.50 (7–153)	27/50	86.230 ± 6.815 (72.874–99.587)	98.000 ± 11.099 (76.246–119.754)	95.7 ± 2.9	85.1 ± 5.2	80.5 ± 5.8	67.7 ± 7.2	
Group 4	Baseline albumin ≥3.5 g/dL Mean albumin <3.5 g/dL	32 (5–178)	48/69	54.501 ± 6.396 (41.965–67.038)	37.000 ± 7.803 (21.707–52.293)	90.8 ± 3.6	71.5 ± 5.7	49.1 ± 6.5	33.8 ± 6.6	

*A value of *p* < 0.05 was considered statistically significant.

**Figure 5.**
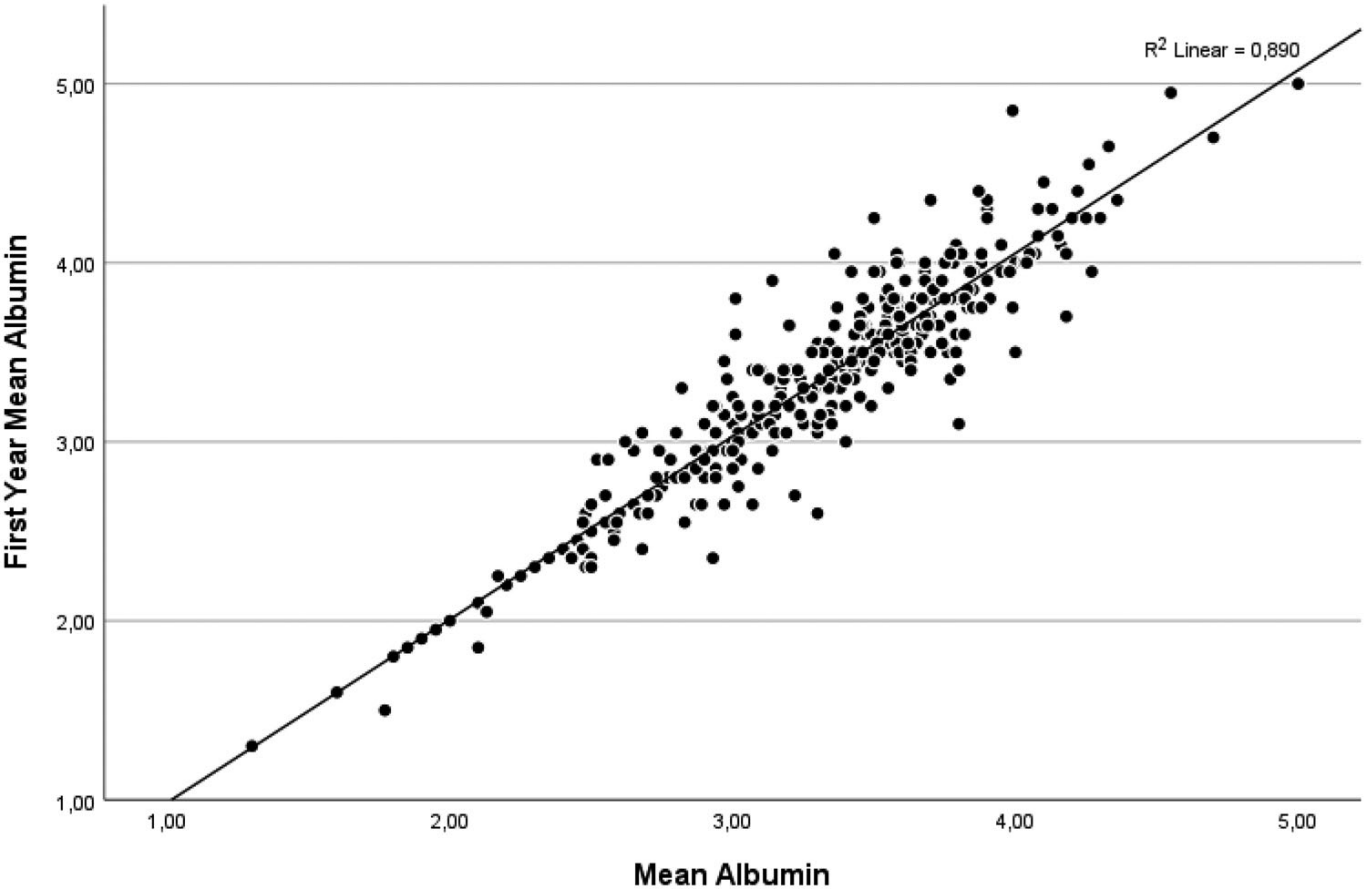
Pearson correlation plot of first year mean albumin and mean albumin values. There was a positive correlation between 1st year mean albumin values and mean albumin values (Pearson = 0.944, *p* < 0.001).

Multivariate Cox regression analysis was performed by modeling to determine the factors affecting mortality in PD patients. Model 1 was performed by comparing basal albumin and mean albumin levels. The mean albumin value was found to have a more significant effect on mortality (HR 1.161 [95% CI 0.229–0.429] (*p* < 0.001) (Table 6)). In the analysis we performed in Model 2, we compared baseline albumin and first-year albumin. We determined that first-year albumin was more predictive of mortality than baseline albumin (HR 0.918 [95% CI 0.302–0.528] (*p* < 0.001) (Table 6)). In addition, age, the presence of urine at the end of peritoneal dialysis, and experiencing peritonitis were factors affecting mortality (Table 6). There was a high positive correlation (Pearson = 0.944, *p* < 0.001) between the 1st year’s mean albumin values and the mean albumin values (Figure 5).

**Table 6.** Multivariate cox proportional hazards regression analysis showing factors affecting mortality.

	*β*	SE*_β_*	Wald	*p*	*eβ* = HR	*eβ* = 95% confidence interval for OR		*E*-value*	
						Bottom	Upper	Effect estimate	Confidence interval limit
Model-1									
Age groups (<65 and ≥65)	0.385	0.141	7.399	0.007*	1.469	1.114	1.939	2.3	1.47
Presence of diabetes mellitus	0.095	0.146	0.426	0.514	1.100	0.826	1.465	1.43	1
Presence of hypertension	−0.085	0.144	0.349	0.554	0.918	0.692	1.218	1.4	1
Presence of coronary artery disease	−0.292	0.169	2.968	0.085	0.747	0.536	1.041	2.01	1
Presence of urine in peritoneal dialysis endpoint	0.568	0.153	13.762	<0.001*	1.764	1.307	2.381	2.92	1.94
Presence of urine reduction in the peritoneal dialysis process	−0.103	0.200	0.264	0.608	0.902	0.609	1.336	1.46	1
Having peritonitis	−0.353	0.172	4.224	0.040*	0.703	0.502	0.984	2.2	1.14
Baseline albumin	−0.081	0.128	0.405	0.525	0.922	0.718	1.184	1.39	1
Mean albumin	−1.161	0.160	52.672	<0.001*	0.313	0.229	0.429	5.84	4.09
Urine volume	0.000	0.000	5.226	0.022*	1.000	0.999	1.000	1	1
Model-2									
Age groups (<65 and ≥65)	0.414	0.140	8.686	0.003*	1.513	1.149	1.992	2.39	1.56
Presence of diabetes mellitus	0.145	0.146	0.990	0.320	1.156	0.869	1.538	1.58	1
Presence of hypertension	−0.025	0.146	0.030	0.862	0.975	0.733	1.297	1.19	1
Presence of coronary artery disease	−0.247	0.169	2.138	0.144	0.781	0.561	1.088	1.88	1
Presence of urine in peritoneal dialysis endpoint	0.614	0.153	16.128	<0.001*	1.847	1.369	2.492	3.1	2.08
Presence of urine reduction in the peritoneal dialysis process	−0.160	0.201	0.631	0.427	0.852	0.575	1.264	1.63	1
Having peritonitis	−0.258	0.171	2.285	0.131	0.773	0.553	1.080	1.91	1
Baseline albumin	−0.083	0.132	0.396	0.529	0.920	0.710	1.192	1.39	1
First-year mean albumin	−0.918	0.142	41.595	<0.001*	0.399	0.302	0.528	4.45	3.2
Urine volume	0.000	0.000	7.160	0.007*	1.000	0.999	1.000	1	1
Model-3									
Age groups (<65 and ≥65)	0.470	0.141	11.166	0.001*	1.600	1.214	2.107	2.58	1.72
Presence of diabetes mellitus	0.094	0.149	0.397	0.529	1.098	0.820	1.470	1.43	1
Presence of hypertension	−0.116	0.145	0.642	0.423	0.890	0.670	1.183	1.5	1
Presence of coronary artery disease	−0.307	0.170	3.263	0.071	0.736	0.527	1.026	2.06	1
Presence of urine in peritoneal dialysis endpoint	0.665	0.150	19.575	<0.001*	1.944	1.448	2.610	3.3	2.25
Presence of urine reduction in the peritoneal dialysis process	−0.230	0.198	1.343	0.247	0.795	0.539	1.172	1.83	1
Having peritonitis	−0.378	0.172	4.857	0.028	0.685	0.489	0.959	2.28	1.25
Albumin groups			33.479	<0.001*					
Group-1	Reference								
Group-2	−0.942	0.196	23.104	<0.001*	0.390	0.266	0.573	4.57	2.89
Group-3	−1.008	0.228	19.505	<0.001*	0.365	0.233	0.571	4.92	2.9
Group-4	−0.325	0.177	3.368	0.066	0.723	0.511	1.022	2.11	1
Urine volume	0.000	0.000	3.910	0.048*	1.000	1.000	1.000	1	1

HR: hazard ratio.

The e-value is the minimum association strength that an unmeasured confounder must have to fully explain a given mortality-outcome association, based on the covariates measured. The larger the e-value, the larger the unmeasured confounder will be needed to explain the predicted association (presented as HR).

E-değeri, ölçülen ortak değişkenlere bağlı olarak, belirli bir mortalite-sonuç ilişkisini tam olarak açıklamak için ölçülmemiş bir karıştırıcının sahip olması gereken minimum ilişki gücüdür. E-değeri ne kadar büyük olursa, tahmin edilen ilişkiyi (HR olarak sunulur) açıklamak için daha büyük ölçülmemiş bir karıştırıcıya ihtiyaç duyulacaktır.

*A value of *p* < 0.05 was considered statistically significant.

## 4. Discussion

Serum albumin level is a biochemical parameter that is affected by protein and calorie intake, dialysis adequacy, peritoneal and renal albumin loss, concomitant inflammation, and underlying systemic diseases. It has proven to be an important determinant of mortality in peritoneal dialysis. Serum albumin levels change dynamically during PD. It has been reported that serum albumin reaches its highest levels after 1 year of PD, regardless of baseline albumin levels [17]. It has been reported that initial serum albumin or single point serum albumin levels, such as steady state and peak phase, at the onset of PD are significantly associated with mortality [5,7,17]. In this study, we determined that the initial serum albumin level alone is not sufficient to determine mortality, and even if the baseline albumin value is low, the increase in albumin values in the PD process will reduce mortality. Again, in this study, we determined that the mean albumin level and the mean albumin level in the first year offered more significant predictions in predicting mortality in the Cox regression analysis compared to the baseline albumin value. Some studies have shown that albumin trajectories after PD are better at predicting mortality than baseline serum albumin [11]. In a different study, it was reported that the time-averaged albumin level predicts a better prognosis than the baseline albumin value [12]. In another study, it was reported that an increase of 0.3 g/dl in serum albumin value significantly reduced mortality [6]. The increase in mortality in patients with normal serum albumin at the onset of PD and decreased mean albumin level or the decrease in mortality in patients with low albumin level at the beginning and normal mean albumin level supports our conclusion. Low baseline albumin may be due to reasons, such as long-term strict protein restriction and decreased appetite in chronic renal failure. In addition to improving gastrointestinal symptoms after the removal of uremic toxins, nutritional intervention may play a role in ameliorating hypoproteinemia after PD. In addition, an increase in albumin levels has been shown to reduce oxidative stress, which is a risk factor for unconventional cardiovascular disease [18].

Although serum albumin and the risk of peritonitis have been extensively studied and it has been proven that low albumin increases the risk of peritonitis, increases in albumin have a protective effect on the risk of peritonitis [17,19,20]. In this study, no significant difference was found between the peritonitis rates of those with hypoalbuminemia and with normal albumin levels compared to baseline albumin. However, when compared with the first year and mean albumin levels, there was a significant increase in peritonitis rates in those with low albumin levels. The possibility of a mechanical link between impaired immune responses due to hypoalbuminemia may underlie the risk of peritonitis. It has been reported that serum albumin reaches its highest level one year after the onset of PD [17]. In some patients, an increase in albumin levels at the onset of PD may reduce the risk of peritonitis due to hypoalbuminemia. However, this risk continues in patients whose albumin does not improve after the onset of PD or in patients who develop hypoalbuminemia. This may explain the high rate of peritonitis, as the mean and first-year mean albumin values were detected in the patient group with prolonged hypoalbuminemia in this study. This may suggest that although baseline albumin is associated with peritonitis risk, it is not as determinative as the mean and first-year mean albumin levels. In addition, peritonitis has been reported to be associated with both mortality and morbidity. The fact that the rate of peritonitis in group 3 was lower than that in group 2 in our study may explain the difference in mortality between these groups.

It is known that serum albumin levels have an important role in maintaining RRF [9]. A decrease in RRF in peritoneal dialysis is associated with both mortality and morbidity [21,22]. It is also possible that a higher serum albumin level maintains renal blood flow, thereby slowing the progression of RRF loss in PD patients [22]. In this study, we report that RRF is interrupted in a shorter time in the group of patients with low mean albumin levels, and RRF continues for longer with the increase in the baseline albumin level.

In conclusion, mean and first-year mean albumin levels provide more predictive predictions for mortality, risk of peritonitis, and maintenance of residual renal functions in peritoneal dialysis patients than baseline albumin.

### 4.1. Study limitations

The strengths of our study included taking routinely collected databases and linking them together, capturing a comprehensive availability of detailed patient-level data. In addition, since the baseline albumin level will be affected by various factors, it gave the average of the 3-month period before the onset of PD. There were some limitations. The sample size was relatively small and included a single-center experience. The study was retrospective with all inherent bias, and the data were limited to what was found in medical charts. Due to the retrospective nature of the study, information on the exact cause of death could not be given. Causes of death could not be analyzed, and therefore, information on multimorbidity could not be given. Nutritional status and malnutrition–inflammation complex syndrome (MICS) evaluation could not be performed since it was a retrospective study. Our study did not include data other than BMI of nutritional status, which is thought to affect serum albumin.

## Data Availability

You can reach Table 1 from the link: https://docs.google.com/document/d/1WXYBx-I5KpLexZqcYCJ_8A4TF40gffHC/edit?usp=sharing&ouid=106512140084758313140&rtpof=true&sd=true.
